# Characterisation of *Roseomonas mucosa* isolated from the root canal of an infected tooth

**DOI:** 10.1186/s13104-017-2538-4

**Published:** 2017-06-14

**Authors:** Nina Diesendorf, Stefanie Köhler, Walter Geißdörfer, Tanja Grobecker-Karl, Matthias Karl, Andreas Burkovski

**Affiliations:** 1 Friedrich-Alexander-Universität Erlangen-Nürnberg, Professur für Mikrobiologie, Staudtstr. 5, 91058 Erlangen, Germany; 2 Friedrich-Alexander-Universität Erlangen-Nürnberg, Universitätsklinikum ErlangenMikrobiologisches Institut–Klinische Mikrobiologie, Immunologie und Hygiene, Wasserturmstr. 3-5, 91054 Erlangen, Germany; 3 Friedrich-Alexander-Universität Erlangen-Nürnberg, Universitätsklinikum Erlangen, Zahnklinik 2, Glückstr. 11, 91054 Erlangen, Germany; 4 Universitätsklinikum des Saarlandes, Klinik für Zahnärztliche Prothetik und Werkstoffkunde, Homburg, Germany

**Keywords:** Antibiotic susceptibility, Biofilm formation, Emerging pathogen, Endodontic infections, Root canal colonization, Systemic infections

## Abstract

**Objective:**

The genus *Roseomonas* comprises a group of pink-pigmented, slow-growing, aerobic, non-fermentative Gram-negative bacteria, which have been isolated from environmental sources such as water and soil, but are also associated with human infections. In the study presented here, *Roseomonas mucosa* was identified for the first time as part of the endodontic microbiota of an infected root canal and characterised in respect to growth, antibiotic susceptibility and biofilm formation.

**Results:**

The isolated *R. mucosa* strain showed strong slime formation and was resistant to most β-lactam antibiotics, while it was susceptible to aminoglycosides, carbapenemes, fluorochinolones, polymyxines, sulfonamides and tetracyclines. Biofilm formation on artificial surfaces (glass, polystyrene, gutta-percha) and on teeth was tested using colorimetric and fluorescence microscopic assays. While solid biofilms were formed on glass surfaces, on the hydrophobic surface of gutta-percha points, no confluent but localised, spotty biofilms were observed. Furthermore, *R. mucosa* was able form biofilms on dentin. The data obtained indicate that *R. mucosa* can support establishment of endodontic biofilms and furthermore, infected root canals might serve as an entrance pathway for blood stream infections by this emerging pathogen.

## Background

Biofilms are groups of sessile microorganisms living within a self-produced matrix of extracellular polymeric substances. These microbial communities are ubiquitously found on abiotic and biotic surfaces including human implants and tissues. In bacterial infections, biofilm formation can significantly increase pathogenicity of bacteria and protection of microorganisms from disinfectants and antibiotics (for a recent overview, see [1]). Mixed biofilm communities are also involved in dental infections, e.g. infections of the root canal [2]. The often complex anatomies of root canals with e.g. isthmuses and lateral canals [3, 4] can cause failure of endodontic therapy due to the persistence of microorganisms in the root canal system and dentin tubules due to insufficient removal of biofilm and disinfection [5]. Beside some prominent species, such as *Enterococcus faecalis* (see e.g. [6]), which are observed routinely, different Gram-negative and Gram-positive bacteria as well as yeasts have already been described as part of the microbiota of infected root canals (see e.g. [7–9]). Many of these microorganisms are only poorly characterised, although they might significantly contribute to persistence of the microbiota. Special properties which might be important in this respect are e.g. the production of antibiotic resistance determinants or extracellular polymer matrices crucial for biofilm formation [10].

In this communication, we describe the characterization of a *Roseomonas mucosa* strain isolated during treatment of an infected root canal. The genus *Roseomonas* comprises a group of pink-pigmented, slow-growing, aerobic, non-fermentative Gram-negative bacteria, which have been isolated from environmental sources such as water and soil, but are also associated with human infections [11–15]. Twenty different species with validly published names were described [16, 17]. Infections of humans with *Roseomonas* species are rare and mainly observed in immunocompromised patients, most likely due to a low pathogenic potential of the bacteria. Catheter-related bloodstream, urinary and respiratory tract infections with different species of the genus were reported [18, 19]. *R. mucosa* seems to be the most prevalent species in clinical samples [18, 20, 21] and skin microbiota seems to be the main reservoir of this species [22]. In contrast to other species, in case of infections with *R. mucosa*, a considerable number of immunocompetent patients have been reported [20, 23] indicating a higher pathogenicity and making the bacterium an emerging pathogen [22].

## Methods

### Sample collection

Samples were collected during regular root canal treatment following informed patient consent and ethics commission approval. Following local anesthesia and isolation of the tooth with rubber dam (Roeko Flexi Dam non latex, Coltene/Whaledent, Langenau, Germany), access cavities to the pulp chambers of the teeth were created using high speed diamond rotary instruments under ambient irrigation. The root canals were subsequently instrumented using sterile C-Pilot files (ISO size 08, 10, 15, VDW, Munich, Germany) which were directly placed in sterile 2 ml test tubes. 2 ml of sterile phosphate-buffered saline was added and the samples were vortexed for 60 s. Subsequently, the buffer was plated-out on different nutrient agar plates (BHI, Columbia Blood Agar, LB, Slanetz and Bartley) obtained from Oxoid (Basingstoke, UK). Arising colonies were streaked-out at least twice to obtain pure cultures, which were used for identification. In summary, pilot files from 13 root canal-treated teeth were investigated, with six showing no bacterial colonization.

### Mass spectrometric identification

A thin layer of bacteria from fresh colonies was spotted on a stainless steel target using a toothpick and overlaid with 1 µl of HCCA matrix (10 mgl α-cyano-4-hydroxycinnamic acid ml^−1^ in 50% acetonitrile/2.5% trifluoro-acetic acid). After drying at ambient temperature identification was performed by MALDI-TOF MS using a Microflex LTTM and the BiotyperTM 3.1 Software (Bruker Daltonik GmbH, Bremen, Germany) on the basis of 240 single spectra.

### Antibiotic susceptibility test

Susceptibility to antibiotics was tested by incubation of bacteria and together with antibiotic disks for 48 h on Mueller–Hinton agar and results were interpreted using the breakpoints for zone diameters of the European Committee on Antimicrobial Susceptibility Testing (EUCAST, http://www.eucast.org). When no breakpoints were available, criteria for related bacteria have been used.

### Biofilm formation

Biofilm formation on artificial surfaces was tested in glass tubes filled with 4 ml BHI and LB medium, respectively. Tubes were incubated on a rotary shaker at 37 °C and biofilm formation was analysed after 1, 3 and 5 days. For this purpose, medium (control) and *R. mucosa* cultures were removed, the tubes were washed twice with water and biofilm staining and quantitative analysis was carried out as described [24]. For colonization of gutta-percha points (Maxima gutta-percha points #30, Henry Schein Dental, Langen, Germany) these were incubated with bacteria in glass tubes (control: incubation in not inoculated medium). Additionally, biofilm formation on polystyrene was tested in microtiter plates [24].

### Fluorescence microscopy of dentin colonization

For fluorescence microscopy, extracted bisected and sterilized teeth were incubated in *R. mucosa*-inoculated BHI medium flasks for 3–4 days. For staining, teeth sections were removed from the flasks, rinsed with distilled water and incubated for 15 min in SYTO9 solution (ThermoFisher Scientific, Dreieich, Germany). Fluorescence microscopic inspection at 20× magnification was carried out using a Zeiss Axio Imager A1 (Carl Zeiss Microscopy GmbH, Oberkochen, Germany) with fixed wavelength and filters for GFP fluorescence equipped with a digital camera (Zeiss Axio Cam MRc5) and analysing software (Zeiss ZEN 2011, version 1.0).

## Results

### Isolation of microorganisms and growth of *Roseomonas mucosa*

**Fig. 1:**
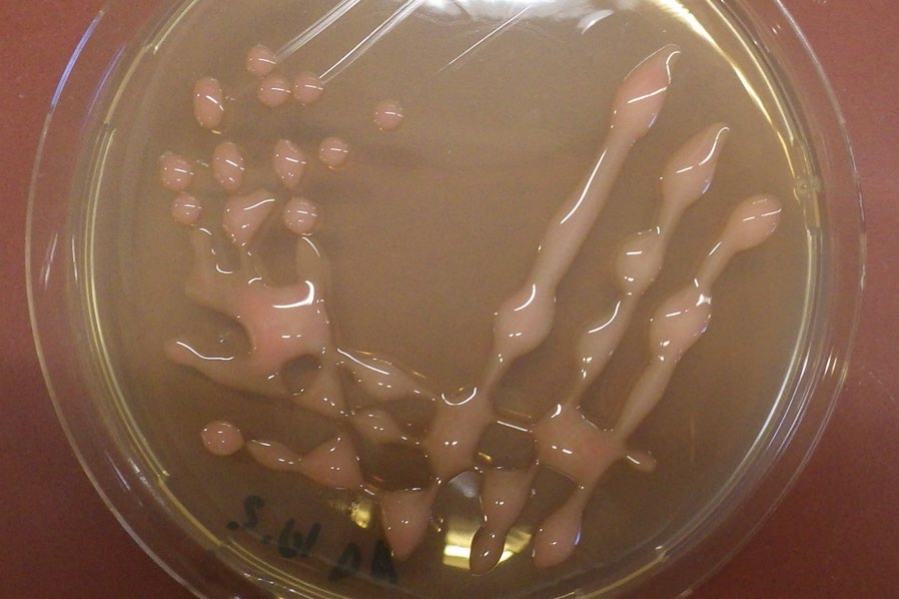
Colonies of *R. mucosa*. Bacteria were streaked-out on LB agar plates and incubated at 37 °C. A strong formation of slime is observed giving the streak-out a runny appearance

In frame of this study, thirteen teeth were tested for microbial colonization of the root canal. In seven cases, microorganisms could be isolated (see Table 1), while six samples were sterile. The majority of isolates were identified as infectors of the human root canal before, e.g. bacteria such as *Actinomyces oris*, *E. faecalis*, *Lactobacillus* species, *Streptococcus oralis* and *Streptococcus sanguinis* or yeasts such as *Candida albicans* and *Candida dubliniensis,*. Among microorganisms isolated from a file used to prepare the root canals of an inflamed tooth, pinkish white pigmented colonies with a mucoid, almost runny appearance were frequently observed. Pure cultures were obtained by careful re-streaking (Fig. 1) and using MALDI-ToF mass spectrometry the corresponding bacteria were identified as *R. mucosa* with a score of 2.4. The bacteria grew on all tested media (Columbia Blood Agar, LB, Slanetz and Bartley), with a preference on rich BHI medium. Slime formation was observed on all solid media tested as well as in liquid culture from which the extracellular polymer could be easily harvested by filtration (data not shown).

**Table 1:** Microbial species isolated from infected root canals

Phylum	Genus	Species
Actinobacteria	*Actinomyces*	*naeslundii*
	*Actinomyces*	*oris*
	*Rothia*	*aeria*
	*Rothia*	*dentocariosa*
	*Corynebacterium*	*durum*
Firmicutes	*Bacillus*	*pumilus*
	*Enterococcus*	*faecalis*
	*Enterococcus*	*faecium*
	*Lactobacillus*	*casei*
	*Lactobacillus*	*paracasei* spp. *paracasei*
	*Lactobacillus*	*plantarum*
	*Lactobacillus*	*Rhamnosus*
	*Staphylococcus*	*Hominis*
	*Streptococcus*	*Oralis*
	*Streptococcus*	*Sanguinis*
Proteobacteria	*Roseomonas*	*Mucosa*
Ascomycota	*Candida*	*Albicans*
	*Candida*	*Dubliniensis*

### Antibiotics resistance

When the isolated strain was tested in respect to antibiotics resistance using the Kirby-Bauer disk diffusion test, *R. mucosa* showed resistance to the β-lactam antibiotics ampicillin, ampicillin/sulbactam, piperacillin and piperacillin/tazobactam as well to the cephalosporines cefazolin, cefuroxime and ceftazidime. Surprisingly and in contrast to the other cephalosporines and penicillines tested, *R. mucosa* was susceptible to ceftriaxone. Sensitivity to the carbapenemes imipenem and meropenem, to the aminoglycosides gentamicin, tobramicin and amikacin and to the fluorochinolone ciprofloxacin was observed, while the strain was resistant to fosfomycin. Sensitivity was also found to the sulfonamides trimethoprim/sulfamethoxazole, the tetracycline antibiotics tetracycline and tigecycline and to polymyxin B (Table 2).

**Table 2:** Susceptibility of *R. mucosa* to antibiotics

Antibiotic	Amount (µg)	Breakpoint (mm)	Susceptibility
β-Lactams (penicillins and cephalosporins)			
Ampicillin	10	6	Resistant
Ampicillin/sulbactam	10/10	6	Resistant
Piperacillin	100	6	Resistant
Piperacillin/tazobactam	100/10	6	Resistant
Cefazolin	30	6	Resistant
Cefuroxime	30	6	Resistant
Ceftriaxone	30	40	Sensitive
Ceftazidime	10	6	Resistant
Carbapenemes			
Imipenem	10	43	Sensitive
Meropenem	10	38	Sensitive
Aminoglycosides			
Gentamicin	10	40	Sensitive
Tobramicin	10	42	Sensitive
Amikacin	30	48	Sensitive
Fluorochinolones			
Ciprofloxacin	5	35	Sensitive
Fosfomycin	200	6	Resistant
Sulfonamide			
Trimethoprim/sulfamethoxazole	1.25/23.75	22	Sensitive
Tetracyclines			
Tetracycline	30	34	Sensitive
Tigecycline	15	37	Sensitive
Polymyxines			
Polymyxin B	300 E	20	Sensitive

Susceptibility to antibiotics was tested by incubation of bacteria together with antibiotic disks for 48 h on Mueller–Hinton agar and results were interpreted using the breakpoints for zone diameters of the European Committee on Antimicrobial Susceptibility Testing (EUCAST, http://www.eucast.org)

### Biofilm formation

**Fig. 2:**
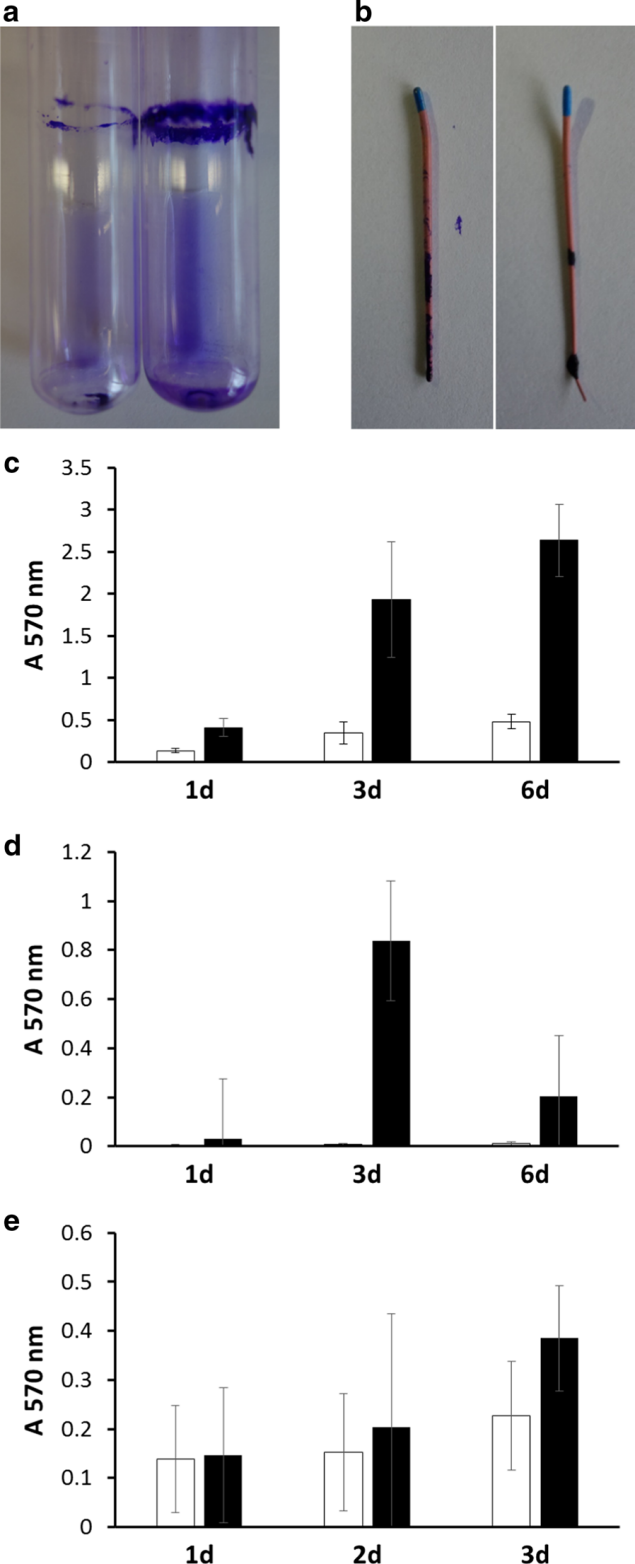
Biofilm formation on artificial surfaces. **a** Image of a* crystal violet*-stained biofilm of *R. mucosa* on the glass surface of a test tube after 6 days of growth. Note the strong stain at the medium-air interphase. **b** Colonization of gutta-percha points. Quantitative analysis of biofilm formation on glass (**c**), gutta-percha (**d**) and polystyrene (**e**) during growth in LB (*white columns*) and BHI (*black columns*) medium. Experiments were carried out at least in three biological replicates and standard deviations are shown

The extremely slimy, almost runny appearance of *R. mucosa* colonies (Fig. 1) indicated a strong formation of extracellular polymers, which may protect the bacteria against dehydration and detrimental environmental conditions. Furthermore, the polymer might support biofilm formation. Different surfaces were tested, starting with established model material such as glass and polystyrene [24] followed by gutta-percha points used as filling material in root canal treatment as well as teeth, and extracellular polymers and sessile bacteria were stained using crystal violet. Independent of the medium and surface material used, biofilm formation was detectable (Fig. 2). *R. mucosa* grew predominantly at the medium-air interphase (Fig. 2a), as it can be expected due to a higher energy yield when oxygen is used as final electron acceptor. A time course of biofilm formation on glass (test tube), gutta-percha and polystyrene (microtiter plate) surfaces and in LB and BHI medium revealed that biofilm formation is coupled to growth of the culture and increases with time.

While glass surfaces were colonized best and stable, solid biofilms were formed (Fig. 2a, c), gutta-percha points and polystyrene surfaces did support only weak attachment of the bacteria. On the surface of gutta-percha points, which comprise a hydrophobic surface, no confluent but local, spotty biofilms were observed (Fig. 2b), which showed high variability and low stability. After 6 days of incubation, previously formed biofilms fell off the gutta-percha points (Fig. 2d). Also biofilms on polystyrene were characterised by only loosely attached material resulting in high standard deviations in quantitative biofilm analyses (Fig. 2e).

**Fig. 3:**
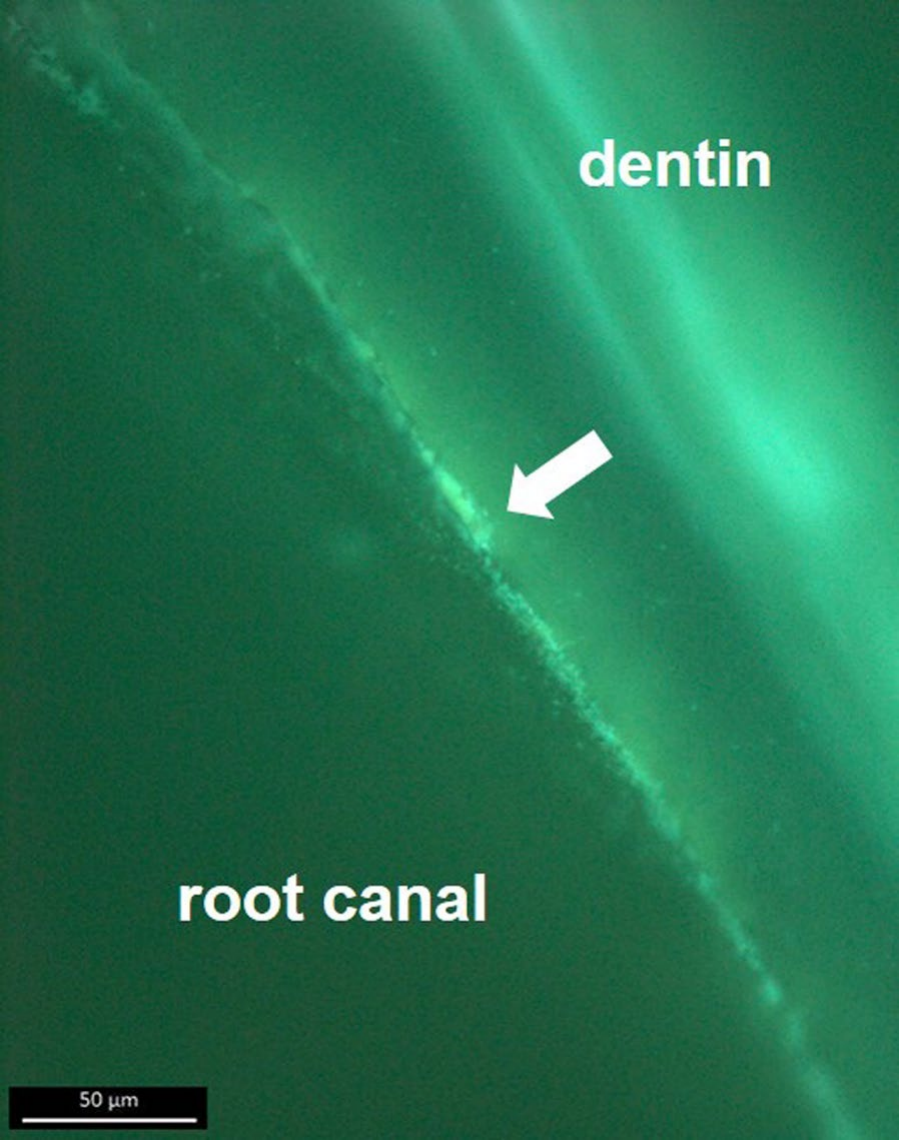
Colonization of dentin. Bisected teeth were incubated for 7 days at 37 °C with *R. mucosa*. Immediately before fluorescence microscopy, teeth were incubated for 15 min with green-fluorescent dye SYTO9. After removal of the fluorescent dye and a washing step with distilled water, bacteria were visualized using a Zeiss AxioCam MRc5 microscope. Bacteria, which appear as *light green spots* embedded in biofilm matrix mainly in the* middle* of the image (indicated by an *arrow*), are predominantly found at the root canal surface, while the dentin shows an even green auto-fluorescence. *Scale bar* 50 µm

Biofilm formation on dentin, the natural surface of the root canal, was tested using extracted teeth. Bacteria were stained with SYTO9, a fluorescent dye penetrating the cell membrane, and visualized by fluorescence microscopy. As shown in Fig. 3, bacteria were colonizing the wall of the root canal in a compact layer of cells and extracellular material.

## Discussion

*Roseomonas mucosa* seems to be the most prevalent *Roseomonas* species in clinical samples and in contrast to other *Roseomonas* species, a considerable number infections of immunocompetent patients has been reported [20, 23]. While previously infections were attributed to environmental sources, a recent study suggest skin microbiota being the main reservoir of this emerging pathogen [22]. The isolation of *R. mucosa* from an infected root canal in this study hint to the possibility that besides catheters also infected teeth might be an entrance pathway for blood stream infections by this pathogen. As indicated by its name, *R. mucosa* is characterised by a strong slime formation, which might be beneficial for biofilm formation. In fact, attachment of the bacteria to different abiotic and biotic surfaces including dentin was found and biofilm material could also be harvested from liquid cultures (data not shown). Hydrophobic surfaces like gutta-percha points seem to obstruct sessile growth of *R. mucosa*.

Biofilms on the root canal wall as found in this study are involved in primary apical periodontitis and may support colonisation of dentinal tubules, which might subsequently contributes to resistance to treatment with disinfectants [2, 5] and antibiotics. *R. mucosa* was susceptible to ceftriaxone, whereas all other cephalosporines and penicillines turned out completely resistant. These results are in accordance with observations made in a recent study by Romano-Bertrand and co-workers ([22]. These authors suggested the production of β-lactamase as reason for resistance. Consequently, the observed resistance to these antibiotics might also protect other pathogenic bacteria from antibiotic treatment when growing together with *R. mucosa* in multispecies biofilms in the host.

## Limitations

This first report of the isolation of *R. mucosa* from an infected root canal and the characterisation of biofilm formation of the corresponding strain might further contribute to the knowledge on this emerging pathogen and its reservoirs. However, further studies besides this single case report and initial experiments are necessary to establish that *R. mucosa* is a significant member of the microbiota of infected root canals and to fully understand its role in oral health.
